# Non-specific Effect of Vaccines: Immediate Protection against Respiratory Syncytial Virus Infection by a Live Attenuated Influenza Vaccine

**DOI:** 10.3389/fmicb.2018.00083

**Published:** 2018-01-31

**Authors:** Young J. Lee, Jeong Y. Lee, Yo H. Jang, Sang-Uk Seo, Jun Chang, Baik L. Seong

**Affiliations:** ^1^Department of Biotechnology, College of Life Science and Biotechnology, Yonsei University, Seoul, South Korea; ^2^Graduate School of Pharmaceutical Sciences, Ewha Womans University, Seoul, South Korea; ^3^Department of Biomedical Sciences, Wide River Institute of Immunology, Seoul National University College of Medicine, Seoul, South Korea; ^4^Vaccine Translational Research Center, Yonsei University, Seoul, South Korea

**Keywords:** non-specific effects, cold-adapted live attenuated influenza vaccine, RSV, innate immunity, cross-protection, toll-like receptor

## Abstract

The non-specific effects (NSEs) of vaccines have been discussed for their potential long-term beneficial effects beyond direct protection against a specific pathogen. Cold-adapted, live attenuated influenza vaccine (CAIV) induces local innate immune responses that provide a broad range of antiviral immunity. Herein, we examined whether X-31ca, a donor virus for CAIVs, provides non-specific cross-protection against respiratory syncytial virus (RSV). The degree of RSV replication was significantly reduced when X-31ca was administered before RSV infection without any RSV-specific antibody responses. The vaccination induced an immediate release of cytokines and infiltration of leukocytes into the respiratory tract, moderating the immune perturbation caused by RSV infection. The potency of protection against RSV challenge was significantly reduced in TLR3^-/-^ TLR7^-/-^ mice, confirming that the TLR3/7 signaling pathways are necessary for the observed immediate and short-term protection. The results suggest that CAIVs provide short-term, non-specific protection against genetically unrelated respiratory pathogens. The additional benefits of CAIVs in mitigating acute respiratory infections for which vaccines are not yet available need to be assessed in future studies.

## Introduction

Respiratory viral infections, caused by influenza virus, respiratory syncytial virus (RSV), human parainfluenza viruses (HPIVs), and coronaviruses, remain a global public health concern (WHO, 2013). WHO has acknowledged the magnitude of this problem and has highlighted the need for research focused on the prevention of respiratory viral infections. However, except for influenza virus, there are no authorized vaccines available for the prevention of emerging/re-emerging respiratory viral infections (Hall, 2001). Vaccination is based on the adaptive immune responses that provide protection against a specific pathogen. Safe and controlled activation of the immune system results in a specific memory in the vaccinated hosts and protects them from secondary infection with the same pathogen (Chang et al., 2014). Recently, several studies have reported non-specific beneficial effects of vaccines on mortality and morbidity; these effects cannot be explained solely by adaptive immunity (Aaby et al., 2014; Goodridge et al., 2016). Growing evidence shows that the innate immune system acquires adaptive characteristics by ‘trained immunity,’ via epigenetic reprogramming of innate immune cells (Blok et al., 2015). A temporary antiviral response could also be established by the turnover of the cells that survived the initial infection (Hamilton et al., 2016). As such, non-specific effects (NSE) of vaccines usually develop a sufficient duration after the initial vaccination. The possibility remains, however, that NSE may also operate in an immediate manner for short-term protection, especially considering the potent antiviral responses of the innate immune system (Takeuchi and Akira, 2009). Such an effect is expected to be quite significant, especially with live attenuated vaccines that mimic natural infections (Seo et al., 2007). For example, live influenza vaccination was found to be associated with an immediate and indirect protection from respiratory illness among children (Piedra et al., 2007).

As the main cause of respiratory infections, influenza virus is responsible for seasonal epidemics as well as occasional pandemics worldwide, with high socioeconomic and medical burden (Turner et al., 2003). Among the various types of vaccines, the cold-adapted, live attenuated influenza vaccine (CAIV) elicits a strong mucosal immune response in the respiratory tracts, providing effective immunity against infection (Renegar et al., 2004). Distinct from the currently licensed FluMist^®^, an alternative and independent CAIV based on the X-31ca backbone has been described (Baez et al., 1980). The genetic basis of attenuation of X-31ca has been characterized (Jang et al., 2016), and various CAIVs against seasonal influenza, H5N1 highly pathogenic avian influenza (HPAI), and the 2009 pandemic H1N1 have been evaluated (Jang et al., 2012, 2013, 2014). The X-31ca-based CAIVs provide an immediate, broad spectrum of protection against heterosubtypic and heterotypic influenza viruses (Seo et al., 2007; Jang et al., 2012).

Here, we examined whether X-31ca provides non-specific immediate cross-protection against RSV. Despite being classified into distinctive viral families (Latiff et al., 2004), RSV and influenza share common features in term of pathogenesis. Both viruses cause acute respiratory infections, and initial infection triggers innate immunity via pattern-recognition receptors (PRRs), including TLR3, TLR7, and retinoic acid-inducible gene I (RIG-I) (Diebold et al., 2004; Guillot et al., 2005; Marr et al., 2013). The activated PRRs induce the production of interferons (IFNs), pro-inflammatory cytokines, and chemokines (Iwasaki and Pillai, 2014). IFN-α/β induces robust antiviral and immunomodulatory responses to interfere with virus replication and spread (Gonzalez-Navajas et al., 2012; Durbin et al., 2013), and IFN-γ is also critical for viral clearance of influenza virus and RSV (Weiss et al., 2010; Eichinger et al., 2015). We found, in a mouse infection model, that early innate immune responses were elicited in the respiratory tracts, restricting RSV replication and ameliorating the rapid increase of the early inflammatory cytokines following RSV challenge. The importance of the TLR3/7-dependent pathway was confirmed by using knockout (KO) mice. Considering previous findings on the long-term effects of influenza infection on subsequent RSV exposure (Walzl et al., 2000), we discuss the immediate NSE of CAIV as an additional benefit of vaccination among infants for the prevention of acute respiratory infections.

## Materials and Methods

### Mice and Ethics Statement

Six-week-old female BALB/c mice were purchased from Orient Bio, Inc. (Seoul, South Korea) and TLR3^-/-^ TLR7^-/-^ mice (BALB/c background) were provided by Kangwon National University, South Korea. Mice were maintained under specific-pathogen-free (SPF) conditions, and animal studies were performed with four mice per group (*n* = 4/group) except for TLR3^-/-^ TLR7^-/-^ mice (*n* = 3/group). Animal experiments were conducted in accordance with the Institutional Animal Care and Use Committee (IACUC) of Yonsei University (IACUC-A-201508-412-02, IACUC-A-201508-422-02, IACUC-A-201511-528-01, IACUC-A-201512-543-02, IACUC-A-201512-572-02, and IACUC-A-201611-468-02).

### Cells and Viruses

Madin-Darby canine kidney (MDCK, ATCC, Manassas, VA, United States) cells were cultured in minimal essential medium (MEM, Hyclone, South Logan, UT, United States) containing 10% fetal bovine serum (FBS, Hyclone). X-31ca (H3N2) was propagated in the allantoic cavity of 11-day-old embryonated eggs and titrated by using the plaque assay using MDCK cells at 33°C. RSV A2 was propagated in HEp-2 cells (ATCC) as previously described (Lee and Chang, 2017), and the virus titer was determined in HEp-2 cells by using the standard plaque assay. Briefly, a 10-fold serial dilution of RSV A2 stock was made in serum-free MEM, and inoculated to HEp-2 cells in 6-well plates. After incubation for 90 min at 37°C, supernatants were discarded and 3 mL of the 1% agarose/growth media mixture was added to each well. After the agar turned solid, plates were incubated for 4 days at 37°C. To visualize plaques, 2 mL of 1% agarose containing 50 μg/mL neutral red was added and incubated for 24 h at 37°C, and then plaques were counted (McKimm-Breschkin, 2004).

### Immunization and Preparation of Samples

Six-week-old female mice were anesthetized by intramuscular infection of alfaxalone (3-α-hydroxy-5-α-pregnane-11, 20-dione, Alfaxan; Jurox, Rutherford, NSW, Australia) before intranasal infection with 50 μL of virus suspension (10^6^ PFU of X-31ca or RSV A2) or intraperitoneal infection with 100 μL of formalin inactivated influenza virus (inactivated X-31ca). Retro-orbital bleeding was performed to collect sera from immunized mice for IgG and IgM antibody titration. Mice were euthanized and bronchoalveolar lavage (BAL) fluids were obtained by washing the airway with 1 mL of PBS. BAL cells and supernatants were then separated by centrifugation. BAL fluids were used for the titration of mucosal IgA antibody and cytokine levels, and the collected BAL cells were used to investigate immune cell recruitment. BAL cells were counted by using a hemocytometer. Whole lungs were homogenized, and the lung supernatants and cells were collected by centrifugation. Lung supernatants were used for the viral titration, and the collected lung cells were used to investigate immune cell recruitment. BAL and lung cells were counted using a hemocytometer.

### Lung Viral Titration

After RSV A2 challenge, mice were euthanized, and a lung single-cell suspension was obtained by passing lung tissue through a 70-μm cell strainer into serum-free MEM. The supernatants were collected by centrifugation and lung viral titration was performed by using a standard plaque assay in HEp-2 cells. The lung viral titer was expressed as PFU/g of lung tissue, and the limit of detection was 100 PFU/g.

### Cytokines and Antibody Levels

The levels of X-31ca, RSV G, or F (Sino Biological, Tongzhou Qu, Beijing, China) protein-specific antibodies were determined by ELISA. Plates were coated with 5 × 10^5^ PFU/well of X-31ca or 50 ng/well of RSV G and F proteins at 4°C overnight. After blocking and washing, plates were incubated with twofold dilutions of sera or BAL fluids for 1 h at room temperature (RT). After washing, the plates were incubated with HRP-conjugated secondary goat anti-mouse IgA, IgG, or IgM antibody (A90-103P, 116P, or 101P, Bethyl, Montgomery, TX, United States) for 1 h at RT, followed by washing and incubation with TMB substrate solution (BD Biosciences, San Jose, CA, United States) for 30 min at RT in the dark. The colorimetric reaction was stopped by adding 2N H_2_SO_4_ solution, and the absorbance was measured at 450 nm using a microplate reader. The concentrations of IFN-α and IFN-β were measured by using ELISA kits (PBL, Piscataway, NJ, United States), and the levels of IFN-γ, IL-6, and TNF-α in BAL fluids were determined by using a Magnetic luminex performance assay multiplex kit (R&D systems, Minneapolis, MN, United States). The experiments were conducted following the manufacturer’s protocol.

### Flow Cytometric Analysis

To analyze immune cell populations, BAL and lung cells were incubated with rat anti-mouse CD16/CD32 (BD Biosciences) blocking antibody for 10 min at RT. Then, the BAL cells were stained with anti-Gr-1 (RB6-8C5, BioLegend, San Diego, CA, United States), anti-Siglec-F (E50-2440, BD Biosciences), Ly6c (AL-21, BD Biosciences), anti-MHCII (M5/114.15.2, eBioscience, San Diego, CA, United States), anti-CD11b (M1-70, BioLegend), anti-CD14 (Sa14-2, BioLegend), anti-CD11c (N418, eBioscience), anti-CD49b (DX5, BioLegend), anti-PDCA-1 (eBio927, eBioscience), DAPI (BioLegend), and anti-CD45 (30-F11, BioLegend) antibodies for 30 min at 4°C in dark. The lung cells were also stained with anti-CD3 (17A2, BioLegend), anti-CD4 (RM4-5, BioLegend), anti-CD8 (53-6.7, BioLegend), and anti-CD45 (30-F11, BioLegend) antibodies for 30 min at 4°C in a dark place. The stained cells were fixed in FACS lysing solution (BD biosciences) and analyzed by BD LSR Fortessa (BD Biosciences). All flow cytometry data were analyzed by Flowjo software (TreeStar Inc., Ashland, OR, United States). The gating strategy for immune cell populations is described in Supplementary Figures S1, S2.

### Statistical Analysis

All values are expressed as the mean of each cohort, and the error bar indicates the standard deviation (SD). Student’s *t*-test was used when comparing two groups. One-way analysis of variance (ANOVA) with Dunnett’s correction was performed for comparing more than two groups. A *P* < 0.05 was considered statistically significant.

## Results

### X-31ca Vaccination Reduces the Pulmonary Viral Titer against RSV Infection

**FIGURE 1:**
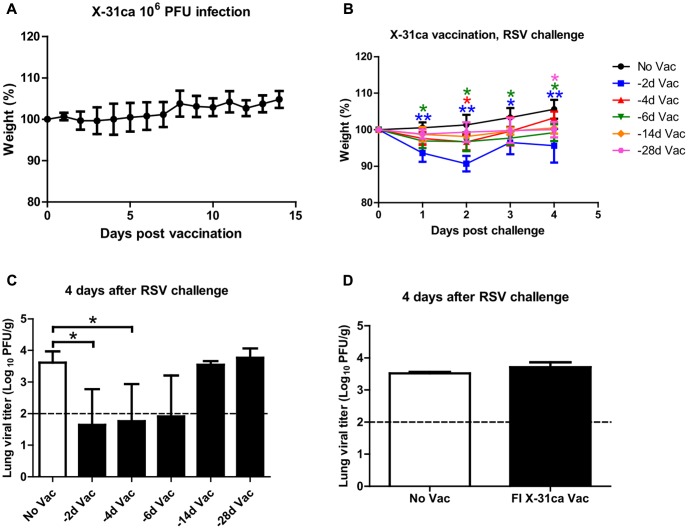
Safety and protective efficacy of X-31ca against RSV challenge. **(A)** Mice (*n* = 4 per group) were vaccinated with 10^6^ PFU of X-31ca and weight changes were observed daily for 2 weeks. **(B,C)** Mice (*n* = 4 per group) were vaccinated with 10^6^ PFU of X-31ca and RSV A2 at 2 (–2d Vac), 4 (–4d Vac), 6 (–6d Vac), 14 (–14d Vac), or 28 (–28d Vac) day intervals. Non-vaccinated (No Vac) mice were infected with 10^6^ PFU of RSV A2 only. The body weight was monitored daily **(B)** and lung viral titer was determined by plaque assay at day 4 p.c. **(C)**. **(D)** Mice (*n* = 4 per group) were vaccinated with formalin-inactivated X-31ca and challenged with 10^6^ PFU of RSV A2 2 days later (FI X-31ca Vac). Non-vaccinated mice were infected with 10^6^ PFU of RSV A2 only (No Vac). Lung viral titer was determined by plaque assay at day 4 p.c. The dotted line represents the limit of detection; 100 PFU/g of lung tissue (*^∗^P* < 0.05, *^∗∗^P* < 0.01 compared with the No Vac control group).

To confirm the safety of CAIV, mice injected with 10^6^ plaque-forming units (PFU) of X-31ca were monitored for any weight change daily for 2 weeks. Infection with X-31ca did not result in any appreciable weight loss as a sign of virulence (**Figure 1A**). Mice were then vaccinated with 10^6^ PFU of X-31ca at various time intervals prior to RSV challenge. Weight changes were observed for 4 days after RSV challenge, and the mice were euthanized at day 4 post-challenge (p.c.) to collect lung tissues. Mice vaccinated with X-31ca 2 days before RSV challenge presented approximately 10% weight loss on day 2 p.c., whereas mice in other groups did not lose body weight (**Figure 1B**). Other indicative signs of illness, such as reduced activity or ruffled fur, were not noticed in the vaccination group on in the control group without vaccination. The lung viral titers dropped below the threshold level upon immunization with X-31ca, preventing RSV replication in the lung for up to 6 days during the interval between immunization and RSV challenge (**Figure 1C**). Pulmonary viral titers gradually increased when the interval was more than 14 days. In contrast, the lung viral titers in the mice vaccinated with formalin-inactivated X-31ca 2 days before RSV challenge were similar to those in unvaccinated mice (**Figure 1D**). RSV titer was measured only in HEp-2 cells, as the lung viral titer from parallel immunization of X-31ca, without RSV challenge, was below the detection level within 4 days of immunization (data not shown). Together, these results demonstrate that X-31ca vaccination could confer early, short-term protection against RSV challenge in mice.

### Early Protection Is Unrelated to Specific Antibody Responses

**FIGURE 2:**
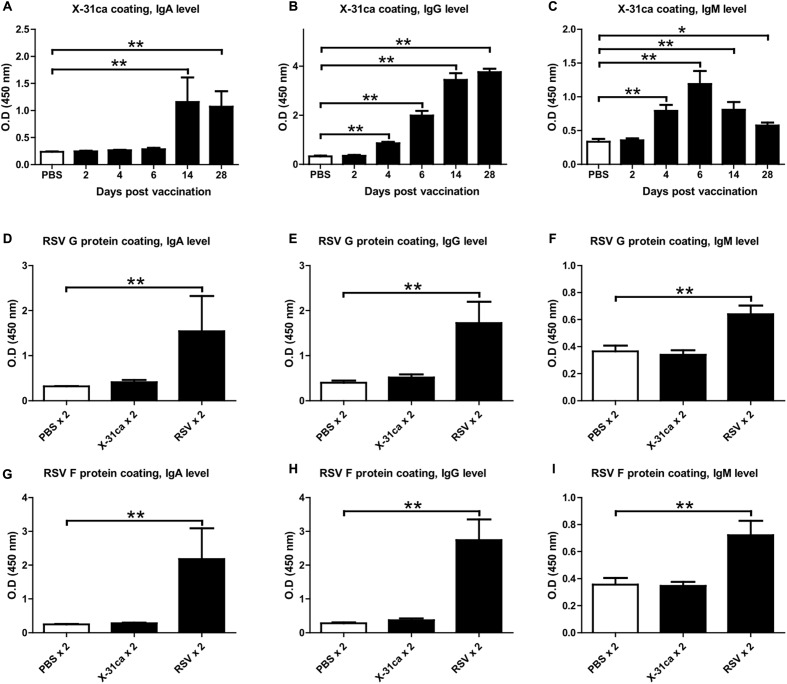
Antibodies from mice vaccinated with X-31ca. **(A–C)** Mice (*n* = 4 per group) were vaccinated with 10^6^ PFU of X-31ca and then euthanized at days 2, 4, 6, 14, and 28 p.v. to collect BAL fluids and sera. ELISAs were performed by using 5 × 10^5^ PFU/well of X-31ca and BAL fluids diluted 1:20 **(A)** or sera diluted 1:320 **(B,C)**. **(D–I)** Mice (*n* = 4 per group) were infected with 10^6^ PFU of X-31ca, RSV A2, or PBS as a negative control and then boosted with the same dose at 2-week intervals. ELISAs were conducted by using 50 ng/well of RSV G protein as the coating antigen and BAL fluids diluted 1:4 **(D)** or sera diluted 1:200 **(E,F)**. ELISAs for RSV F protein (50 ng/well) were performed with BAL fluids diluted 1:4 **(G)** or sera diluted 1:400 **(H,I)**. OD, optical density (*^∗^P* < 0.05, *^∗∗^P* < 0.01 compared with the PBS control group).

The antibody levels from the mice immunized with X-31ca were determined. After vaccination with 10^6^ PFU of X-31ca, BAL fluids and sera were obtained from mice at various days post-vaccination (p.v.). The levels of IgA derived from BAL fluid, and IgG and IgM derived from serum and were analyzed by ELISA for their specificity to X-31ca (**Figures 2A–C**). IgA level against X-31ca was below the detection level within 6 days p.v. and increased rapidly after 14 days (**Figure 2A**). IgG level was detected as early as 6 days and increased rapidly thereafter (**Figure 2B**). IgM antibodies were rapidly produced and peaked on day 6 p.v. (**Figure 2C**). Mice were then immunized with X-31ca twice at 2-week intervals to acquire the X-31ca antibodies. In the ELISA, BAL fluids and sera collected from the X-31ca-injected mice (X-31ca × 2 group) presented OD values similar to those of the control group (PBS × 2 group) against the RSV G and F proteins as the major protective antigens (**Figures 2D–I**). In contrast, mice infected with RSV (RSV × 2 group) on the same schedule showed strong reactivity against RSV G and F proteins. Of note, vaccination with X-31ca prior to infection with RSV did not appreciably change the CD4 and CD8 T cell composition in the lungs (Supplementary Figure S3). The lack of cross-reactivity between X-31ca and RSV even after boost immunization suggests that the immediate protection provided by X-31ca is not antibody-mediated.

### X-31ca Stimulates Innate Immune Responses

**FIGURE 3:**
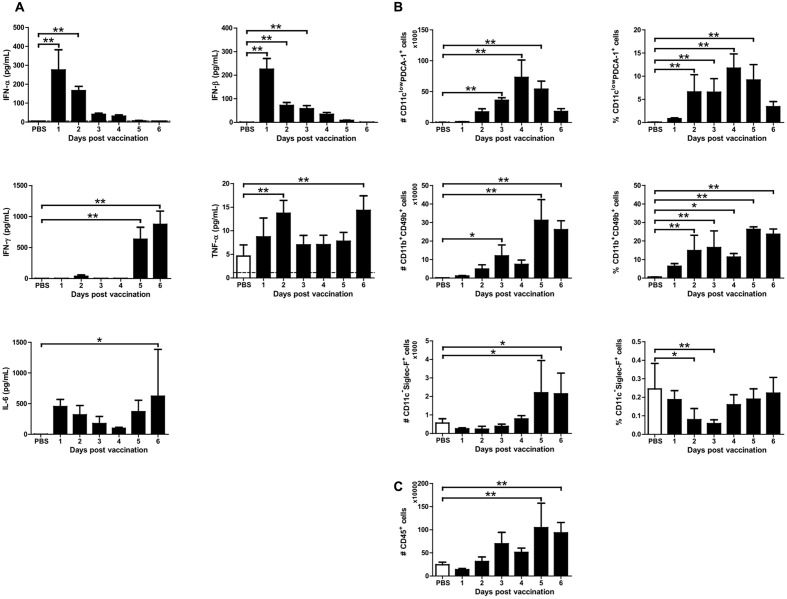
Cytokine levels and immune cell composition after X-31ca vaccination. **(A–C)** Mice (*n* = 4 per group) were vaccinated with 10^6^ PFU of X-31ca, and BAL fluids were collected daily for 6 days. In BAL fluids, the mean concentration of IFN-α and IFN-β was determined by ELISA and the levels of IFN-γ, IL-6, and TNF-α were measured by Luminex **(A)**. The dotted line represents the limit of detection; IFN-α: 5, IFN-β: 0.5, IFN-γ: 3.49, TNF-α: 1.14, IL-6: 2.23 pg/mL, respectively. To investigate the immune cell subsets, cells obtained from BAL were stained with fluorescence antibodies as presented in the Methods and analyzed by flow cytometry **(B,C)** (*^∗^P* < 0.05, *^∗∗^P* < 0.01 compared with the PBS control group).

Because mice vaccinated with X-31ca were protected from RSV challenge without specific antibody responses, we hypothesized that the prophylactic effect was associated with the innate immune response (Seo et al., 2007). BAL fluids and cells were obtained daily for 6 days from mice infected with 10^6^ PFU of X-31ca. The samples were used to examine the levels of IFNs (IFN-α, IFN-β, and IFN-γ) and pro-inflammatory cytokines (TNF-α and IL-6), and the numbers of immune cells (pDCs, NK cells, and eosinophils) as markers for innate immune responses (**Figure 3**). The concentrations of IFN-α and IFN-β peaked on day 1 p.v. and decreased gradually, whereas the induction of IFN-γ was delayed, with its level peaking on day 6 p.v. (**Figure 3A**). Immunization with X-31ca induced lower, but still distinctly elevated, levels of TNF-α and IL-6. Populations of various immune cell types were examined by fluorescence-activated cell sorting (FACS) analysis (**Figures 3B,C**) for both the number of cells (left side) and the percentage of total immune cells (right side). Vaccinated mice elicited more pDCs (CD11c^low^ PDCA-1^+^ cells) than the control group throughout the 6 days of examination, with peak values on days 3 and 4 p.v. (**Figure 3B**). The recruitment of NK cells (CD11b^+^ CD49b^+^ cells) and eosinophils (CD11c^-^Siglec-F^+^ cells) was slightly delayed, showing a peak level on day 5 p.v. The number of CD45^+^ immune cells in BAL was greatly increased as compared with the PBS control after day 3 p.v. (**Figure 3C**). pDCs and NK cells showed similar patterns for both the number and percentage of total immune cells, whereas eosinophils presented a different pattern (**Figure 3B**). This result probably reflected the highest increase in total immune cells on day 5 and 6 p.v. (**Figure 3C**). The number and the proportion of AMs (CD11c^+^Siglec-F^+^ cells) and neutrophils (CD11c^inter^Gr-1^high^ cells) were also examined (described in **Figure 3** and Supplementary Figure S4). However, X-31ca vaccination did not show a significant difference as compared with the control. Overall, the X-31ca vaccination induced distinct marker cytokines and immune cells associated with innate immunity.

### X-31ca Vaccination Changes the Cytokine Profile and the Immune Cell Composition in RSV-Challenged Mice

**FIGURE 4:**
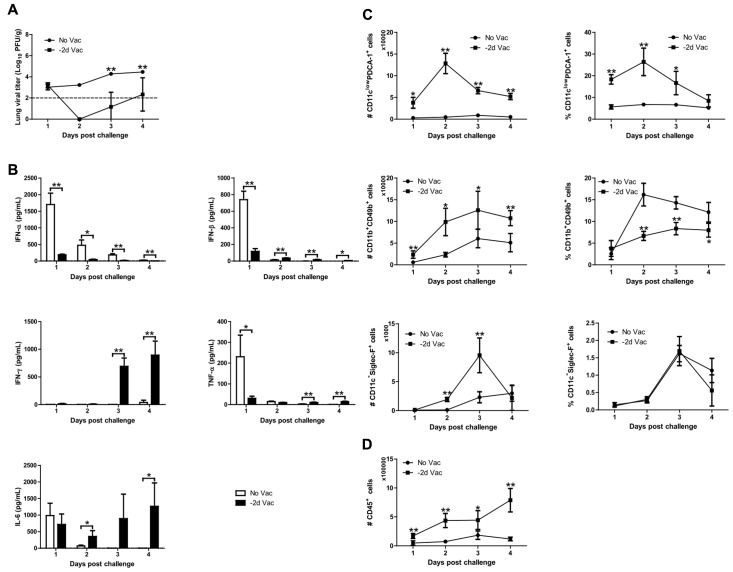
Viral replication, cytokine profile, and immune cell composition. **(A–D)** Mice (*n* = 4 per group) were vaccinated with 10^6^ PFU of X-31ca and challenged with 10^6^ PFU of RSV A2 2 days later (–2d Vac). Non-vaccinated mice were infected with 10^6^ PFU of RSV A2 only (No Vac). After RSV challenge, lungs and BAL fluids were obtained daily for 4 days, and the lung viral titer was determined by plaque assay **(A)**. In BAL fluids, the levels of IFN-α and IFN-β were determined by ELISA, and the mean concentration of IFN-γ, IL-6, and TNF-α was measured by Luminex **(B)**. The dotted line represents the limit of detection; IFN-α: 5, IFN-β: 0.5, IFN-γ: 3.49, TNF-α: 1.14, IL-6: 2.23 pg/mL, respectively. To investigate the immune cell subsets, flow cytometry analysis of cells obtained from BAL was performed as in **Figures 3B,C** **(C,D)** (*^∗^P* < 0.05, *^∗∗^P* < 0.01 compared with the No Vac control group).

To examine the cause for the reduction in RSV replication following X-31ca vaccination (**Figure 1C**), mice were inoculated with X-31ca followed by RSV challenge at 2 days p.v. BAL fluids and lung samples were collected daily for 4 days after RSV challenge and were examined for lung viral titers, cytokine levels, and immune-cell subsets (**Figure 4**). As expected, in contrast to the non-vaccinated control mice, which presented extensive viral growth (>3 × 10^4^ PFU/g), RSV failed to replicate in the lungs of X-31ca-vaccinated mice (**Figure 4A**). Vaccination induced dramatic changes in the cytokine profiles. Notably, the levels of IFN-α, IFN-β, and TNF-α that were strongly elevated by RSV infection were greatly reduced by vaccination (**Figure 4B**). In contrast, IFN-γ levels, which remained extremely low after RSV infection, were strongly elevated by vaccination. Moreover, the IL-6 level was maintained up to 4 days, in contrast to a sharp decrease (∼1 day) in the non-vaccinated mice. Thus, vaccination with X-31ca prior to infection with RSV dramatically changed the cytokine profiles and suppressed RSV infection. It should be noted that days 1–4 after RSV challenge (**Figure 4B**) corresponds to days 3–6 after X-31ca vaccination (**Figure 3A**), considering the 2-day interval between vaccination and challenge. Remarkably, the cytokine profiles and levels in X-31ca-vaccinated mice were relatively constant without notable perturbation following infection with RSV.

Populations of various immune cell types were also examined by FACS analysis (**Figures 4C,D**). The numbers of most immune cells, i.e., pDCs (CD11c^low^ PDCA-1^+^ cells) and NK cells (CD11b^+^ CD49b^+^ cells), were consistently higher in vaccinated mice than in unvaccinated mice, with the exception of eosinophils (CD11c^-^Siglec-F^+^ cells) at days 1 and 4 p.c. (**Figure 4C**). However, NK cells and eosinophils showed different patterns for both the number and proportion of total immune cells, probably reflecting the increase in total immune cells upon vaccination with X-31ca (**Figure 4D**). The number and proportion of AMs (CD11c^+^Siglec-F^+^ cells) and neutrophils (CD11c^inter^Gr-1^high^ cells) were also examined (as described in **Figure 4** and Supplementary Figure S5). The number and percentage of AMs were higher in vaccinated mice than in unvaccinated mice, only at day 2 p.c. In vaccinated mice, the number of neutrophils was consistently higher than that in unvaccinated mice, but the proportion did not show a significant difference between two groups. These results show that X-31ca vaccination recruits a large number of immune cells into the respiratory tract, and stabilizes the immune environment sufficiently to overcome immune perturbation by subsequent infection. The maintenance of antiviral status by X-31ca immunization is probably responsible for the successful inhibition of RSV, stabilizing against immune aggravation by RSV infection.

### Protective Efficacy in TLR3^-/-^ TLR7^-/-^ Mice

**FIGURE 5:**
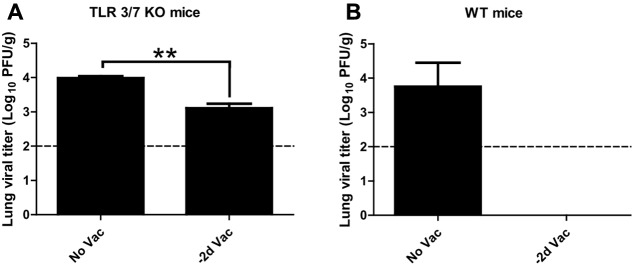
Protective efficacy in TLR3^-/-^ TLR7^-/-^BALB/c mice vaccinated with X-31ca after RSV challenge. **(A)** TLR3/7 knockout (KO) mice (*n* = 3 per group) or **(B)** wild-type (WT) mice (*n* = 3 per group) were vaccinated with 10^6^ PFU of X-31ca and challenged with 10^6^ PFU of RSV A2 2 days later (–2d Vac). Non-vaccinated animals were infected with 10^6^ PFU of RSV A2 only (No Vac). Lung viral titer was determined by plaque assay using lung supernatants obtained from the mice euthanized at day 4 p.c. The dotted line represents the limit of detection; 100 PFU/g of lung tissue (*^∗∗^P* < 0.01 compared with the No Vac control group).

To further investigate the contribution of innate immunity to early protection, transgenic mice deficient in both TLR3 and TLR7 were employed (Diebold et al., 2004; Guillot et al., 2005). TLR3 and TLR7 double knockout (TLR3/7 KO) mice were immunized with X-31ca and infected with RSV at 2 days p.v., and then the level of RSV replication in the lung was measured (**Figure 5A**). As a control, wild type (WT) mice were treated under the same conditions (**Figure 5B**). Whereas X-31ca vaccination caused complete viral clearance in the lung of RSV-infected WT mice, RSV replication persisted in TLR3/7 KO mice despite vaccination with X-31ca. Together, these results suggest that TLR3- and TLR7-mediated innate immunity plays an important role in the early protection against RSV infection using X-31ca.

## Discussion

Several reports have discussed the non-specific beneficial effects of vaccination on mortality and morbidity associated with unrelated infections (Aaby et al., 2014). Trained innate immunity has been proposed as an underlying mechanism for the long-term, NSEs of vaccines (Netea et al., 2011; Blok et al., 2015). Considering the substantial reduction in all-cause mortality with live attenuated vaccines, e.g., oral polio vaccine (OPV) and measles vaccines, the WHO has emphasized the need for more studies on the NSEs of vaccines (Author, 2014; Mina et al., 2015). However, potential short-term effects of vaccination have not been explored to date. In a previous study, immediate and indirect protection from respiratory illness was observed as part of the clinical evaluation of live influenza vaccination among children (Piedra et al., 2007). Here, we hypothesized that the strong innate immune responses elicited by live attenuated vaccines may contribute to non-specific, short-term protection from unrelated pathogens.

In this study, we evaluated the innate immune responses induced by X-31ca and their role in protecting against genetically unrelated RSV infection in a mouse infection model. X-31ca provided immediate protection against RSV infection in an antibody-independent manner in the mouse model. X-31ca vaccination stimulated the production of marker cytokines over the 6 days of examination, recruiting relevant immune cells. Cytokine profiles and immune cells were relatively unperturbed and were maintained even after RSV challenge. Experiments with KO mice confirmed that the innate immunity mediated by TLR3 and TLR7 was responsible for RSV protection. These results demonstrate that X-31ca administration prior to RSV challenge activated a much milder, but distinct, induction of cytokines and recruited more immune cells, and that these innate immune responses conferred early protection against RSV infection. Previously, influenza infection-mediated protection from RSV was evaluated in mice, and a long-lasting influence on subsequent responses to an additional infection was observed (Walzl et al., 2000). In contrast, the present analyses address the immediate response rather than a prolonged and delayed response upon influenza vaccination rather than infection. The present results in both trained and naïve innate immune arms suggest that an indirect, non-specific protection operates on a wide spectrum.

Innate immunity plays an important role in initiating a primary defense mechanism and, later, in adaptive immunity (Takeuchi and Akira, 2009). Notably, the X-31ca-based 2009 H1N1 pandemic vaccine provided heterologous as well as heterosubtypic protection against H5N1 and seasonal influenza (Jang et al., 2012). In addition to the role of adaptive immune responses for broad protection, the X-31ca master strain conferred immediate and broad-spectrum protection against heterotypic influenza B infection (Seo et al., 2007). Previously, prolonged pro-inflammatory cytokine expression after influenza vaccination and an impact on both innate and adaptive responses were noted for live attenuated influenza vaccines (LAIVs) (Lanthier et al., 2011; Mohanty et al., 2015). In this study, we showed that innate responses were potent enough to provide indirect protection against immunologically unrelated RSV infection, albeit within a narrow window of vaccination and infection (**Figure 1C**).

The IgA and IgG antibody levels for X-31ca were very low within 6 days after vaccination (**Figures 2A,B**). In contrast, a high level of IgM response was observed within 6 days after vaccination (**Figure 2C**). The responses against RSV surface proteins (G and F proteins) were as low as the PBS control, in contrast to robust response to X-31ca virus (**Figures 2D–I**). The lack of cross-reactivity between influenza and RSV antigen suggests that the observed cross-protection is not antibody-mediated. Cross-reactive and antigen-non-specific T cell immunity appears to not contribute to the immediate defense provided by X-31ca, as the vaccination with X-31ca prior to infection with RSV did not alter the compositions of CD4 and CD8 cells in the lungs (Supplementary Figure S3).

The vaccination elevated the production of marker cytokines compared to the levels in negative-control mice (**Figure 3A**), but the levels of type I IFN and TNF-α were significantly lower than those in RSV-infected mice (**Figure 4B**). Although the threshold concentration required for inhibition of viral replication was difficult to assess, the cytokine levels produced by X-31ca vaccination were sufficient to clear RSV in the lung (**Figure 4A**). Notably, RSV challenge did not significantly alter the cytokine profiles of X-31ca-vaccinated mice (**Figures 3A**, **4B**), suggesting that immunization activates and maintains a mild antiviral state in mice and protects them from acute perturbation of cytokine levels induced by RSV challenge. A proper innate response leads to viral clearance, whereas imbalanced responses can result in acute respiratory distress (Han and Ulevitch, 2005). Thus, prior vaccination with X-31ca stably maintains the immune environment in mucosal areas, alleviating pathologies such as organ dysfunction caused by over-reactive inflammatory responses induced by respiratory infection.

The immunization recruited more leukocytes, and immune cells such as pDC and NK cells in the respiratory tracts were directly associated with RSV clearance (**Figures 3B**, **4C**). Although the vaccination with X-31ca alone did not affect the composition of AMs (Supplementary Figure S4), the number of AMs also increased when X-31ca and RSV were inoculated at 2-day intervals (Supplementary Figure S5). These cells, which are associated with innate and adaptive immunity (Colonna et al., 2004), were mobilized to the nasal mucosa during RSV infection (Gill et al., 2005). In particular, the pDC number and percentage were elevated in vaccinated mice compared with the unvaccinated control, consistent with the role of pDCs in both virus clearance and prevention of excessive immunopathology (Smit et al., 2006; Wang et al., 2006). These results suggest that the X-31ca immunization-induced increase in pDCs plays a crucial role in viral clearance.

In addition to the pDCs mentioned above, IFN-γ and NK cells are also expected to be key components in the innate immunity that inhibits RSV replication. A direct antiviral effect mediated by IFN-γ could attenuate RSV replication (Wheelock, 1965; Bukreyev et al., 1999). The RSV challenge did not significantly alter the cytokine profiles of X-31ca-vaccinated mice (**Figures 3A**, **4B**), and IFN-γ was highly induced and maintained for several days after X-31ca vaccination (**Figure 4C**), thereby playing an important role in the short-term viral clearance. Moreover, NK cells are associated with viral clearance in the early phase of RSV infection (Harker et al., 2010), and probably also played a role in immediate protection in this study, as reflected in the increase of NK cells by vaccination (**Figure 4C**). More detailed studies, e.g., with KO mice or by depletion of specific immune cells, are required to identify the major components involved in these responses.

In respiratory epithelial cells, the TLR3 signaling pathway directly contributes to innate immunity against influenza virus (Guillot et al., 2005). We previously demonstrated that a TLR3 agonist alone induced a protective immune response and, in combination with a TLR7 agonist, achieved immediate and complete protection against lethal influenza infection (Seo et al., 2007). Vaccination of TLR3/7 KO mice with X-31ca failed to inhibit RSV replication (**Figure 5A**), further confirming that innate immune responses linked with the TLR3/7 pathway are responsible for the cross-protection against RSV. Notably, RSV replication was elevated but not fully recovered in vaccinated mice despite TLR3 and TLR7 deficiency (**Figure 5A**). Other PRRs such as RIG-I (Koyama et al., 2007) may still operate in TLR3/7 KO mice, partially compensating for the impaired innate immunity. Clearly, innate immune responses stimulated by CAIVs can protect the mucosal respiratory tract from RSV infection.

The potential safety issues of vaccination merit further discussion. As reflected in the lung viral titer, RSV replication was significantly inhibited by prior inoculation of X-31ca within 6 days (**Figure 1C**). The observed inhibition was achieved without significant changes in weight loss over 4–6 days. However, noticeable changes in weight loss (up to 10%) were apparent for shorter intervals (2 days) (**Figure 1B**), despite enhanced inhibition of RSV (**Figure 1C**). Previously, it was shown that increased recruitment of NK cells is involved in not only viral clearance but also weight loss (Harker et al., 2010). However, the apparent adverse effect of the increase in NK cell population (**Figure 4C**) could be counter-balanced by the potential beneficial effects associated with the changes in the cytokine profiles. For instance, the decrease in type I IFN by prior vaccination (**Figure 4B**) may reduce its potential adverse effect of increasing the susceptibility to secondary bacterial infections (Li et al., 2012). Prior vaccination with X-31ca **(-2d)** before RSV challenge transiently increased the number of eosinophils and neutrophils (**Figure 4C** and Supplementary Figure S5), which may be responsible for a complex inflammatory response underlying the observed weight loss in the same vaccination/challenge schedule (**Figure 1B**). The generalizability of these findings are limited, however, by the lack of a relevant animal model for influenza and RSV superinfection.

The present results are based on the X-31ca attenuated strain, which has been used for generation of CAIVs for seasonal, pre-pandemic, and potentially universal vaccines (Seo et al., 2007; Jang et al., 2012, 2013, 2014, 2016). It should be noted that, FluMist^®^, as a prototype CAIV, has been licensed for vaccination of individuals > 2 years of age. Most of the genetic mutations in CAIVs that attenuate of virulence are in internal genes (Snyder et al., 1988; Klimov et al., 1992; Jang et al., 2016), but CAIVs could be further attenuated by additional mutations either on surface antigens (Lee et al., 2016) or in internal genes (Liedmann et al., 2014), which could improve the safety profile of CAIVs for infants. A major distinction between the two attenuated strains is that FluMist^®^ is based on the H2N2 strain (Baez et al., 1980; Maassab and DeBorde, 1985), whereas X-31ca is based on the H1N1 backbone (Lee et al., 2006). It would be interesting to determine, therefore, whether similar non-specific protection is provided by the licensed CAIV vaccine. Currently, the possibility of prime-boost-type early childhood vaccination is being debated with a view to implement a universal vaccine program (Krammer and Palese, 2014). With the future prospect of using CAIVs for infants, subject to safety issues associated with live vaccines, additional benefits may be anticipated, especially for non-specific protection from RSV and other respiratory viruses such as HPIVs, for which no licensed vaccines are available (Hall, 2001).

The present studies are based on a mouse model, and present potential limitations such as the lack of a common animal infection model among different virus families. It would be interesting to determine whether the observed non-specific protection could be further extended to a repertoire of respiratory viruses including MERS-coronavirus (MERS-CoV), which recently posed a global public threat (Breban et al., 2013; Choi, 2015). Moreover, most of the NSEs of vaccines have been evaluated in the clinical setting and monitored over the long-term (Kandasamy et al., 2016). Independent studies on the short-term NSEs of vaccinations would be impractical in the clinical setting, but could be implemented during the evaluation of the clinical effectiveness of vaccines (Piedra et al., 2007). The significance of NSEs observed in experimental animal infection remains to be explored further.

## Author Contributions

YL, JL, and BS designed the study, analyzed the data, and wrote the manuscript; YL and JL conducted the research study; YJ and S-US provided technical assistance about CAIV and KO mice, respectively; JC provided the scientific advice about RSV infection and protection; BS supervised all the experiments as well as the preparation of the manuscript.

## Conflict of Interest Statement

The authors declare that the research was conducted in the absence of any commercial or financial relationships that could be construed as a potential conflict of interest.
