# Supplementary material for: Non-specific Effect of Vaccines: Immediate Protection against Respiratory Syncytial Virus Infection by a Live Attenuated Influenza Vaccine
Source: Front Microbiol. 2018 Jan 31;9:83. doi: 10.3389/fmicb.2018.00083 (PMC5797773; doi:10.3389/fmicb.2018.00083)
Supplement: Supplementary file 1 [file Presentation_1.pdf]

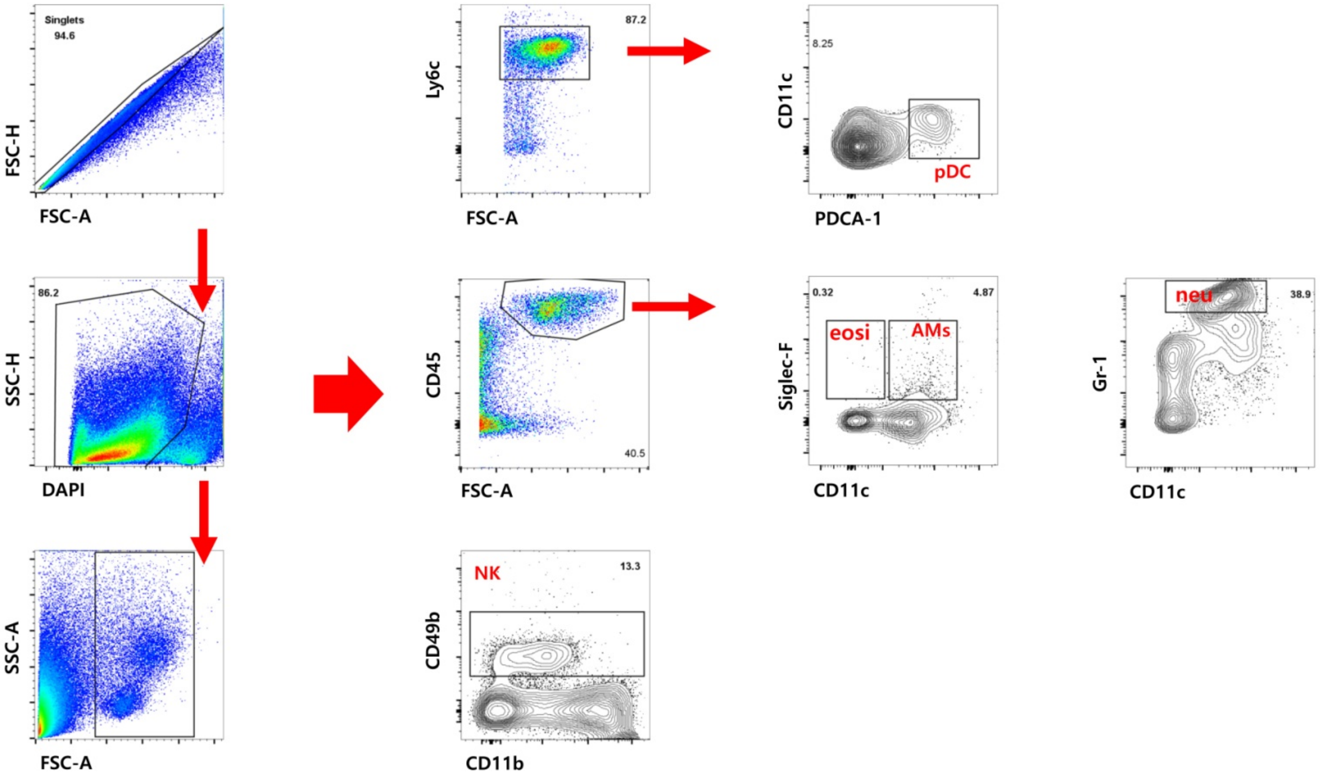

Supplementary Figure S1. Flow cytometry gating strategy for analysis of immune cell populations in BAL. BAL cells were gated for singlets (FSC-H/FSC-A), live cells (SSC-A/DAPI$^-$), and leukocytes (SSC-A/FSC-A). Then, BAL cells were discriminated as follows: Plasmacytoid DCs (pDCs) (Ly6C$^+$/PDCA-1$^+$/CD11c$^-$), eosinophils (CD45$^+$/CD11c$^-$/Siglec-F$^+$), alveolar macrophages (CD45$^+$/CD11c$^+$/Siglec-F$^+$) (Schneider et al., 2014), neutrophils (CD45$^+$/CD11c^{inter}/Gr-1^{high}), and NK cells (CD11b$^+$/CD49b$^+$).

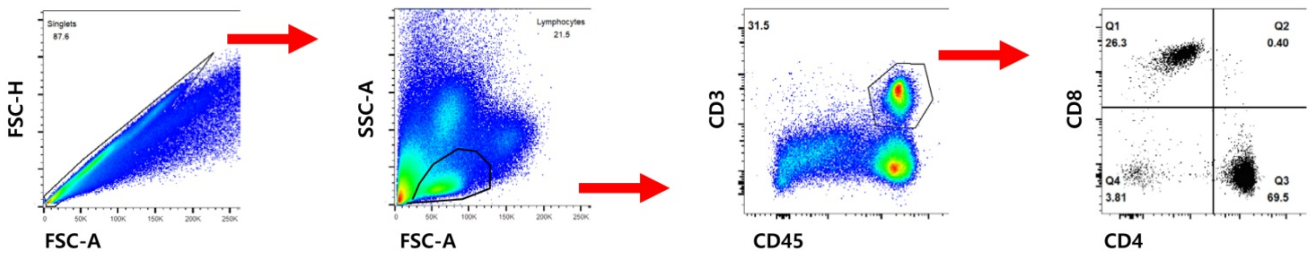

Supplementary Figure S2. Flow cytometry gating strategy for analysis of immune cell populations in the lung. Lung cells were gated for singlets (FSC-H/FSC-A), lymphocytes (SSC-A/FSC-A), and T cells ($CD3^+/CD45^+$). Then, $CD4^+$ and $CD8^+$ T cells were divided.

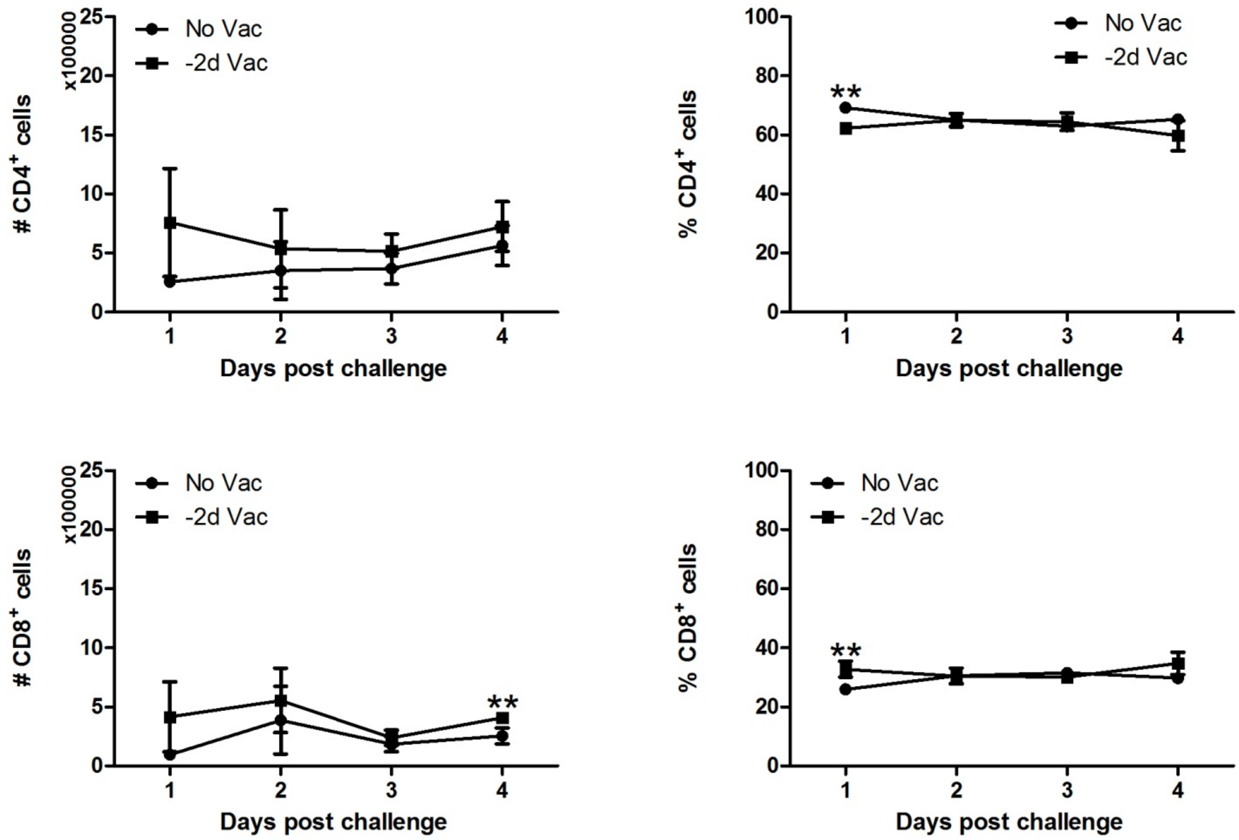

Supplementary Figure S3. CD4 and CD8 T cell immunity. Mice (n=4 per group) were vaccinated with 10^6 PFU of X-31ca and challenged with 10^6 PFU of RSV A2 2 days later (-2d Vac). Non-vaccinated mice were infected with 10^6 PFU of RSV A2 only (No Vac). After RSV challenge, lungs were obtained daily for 4 days. To investigate CD4 and CD8 T cells subsets, cells obtained from lungs were stained with fluorescence antibodies as presented in the Methods and analyzed by flow cytometry. ($^{**}P < 0.01$ compared with the No Vac control group).

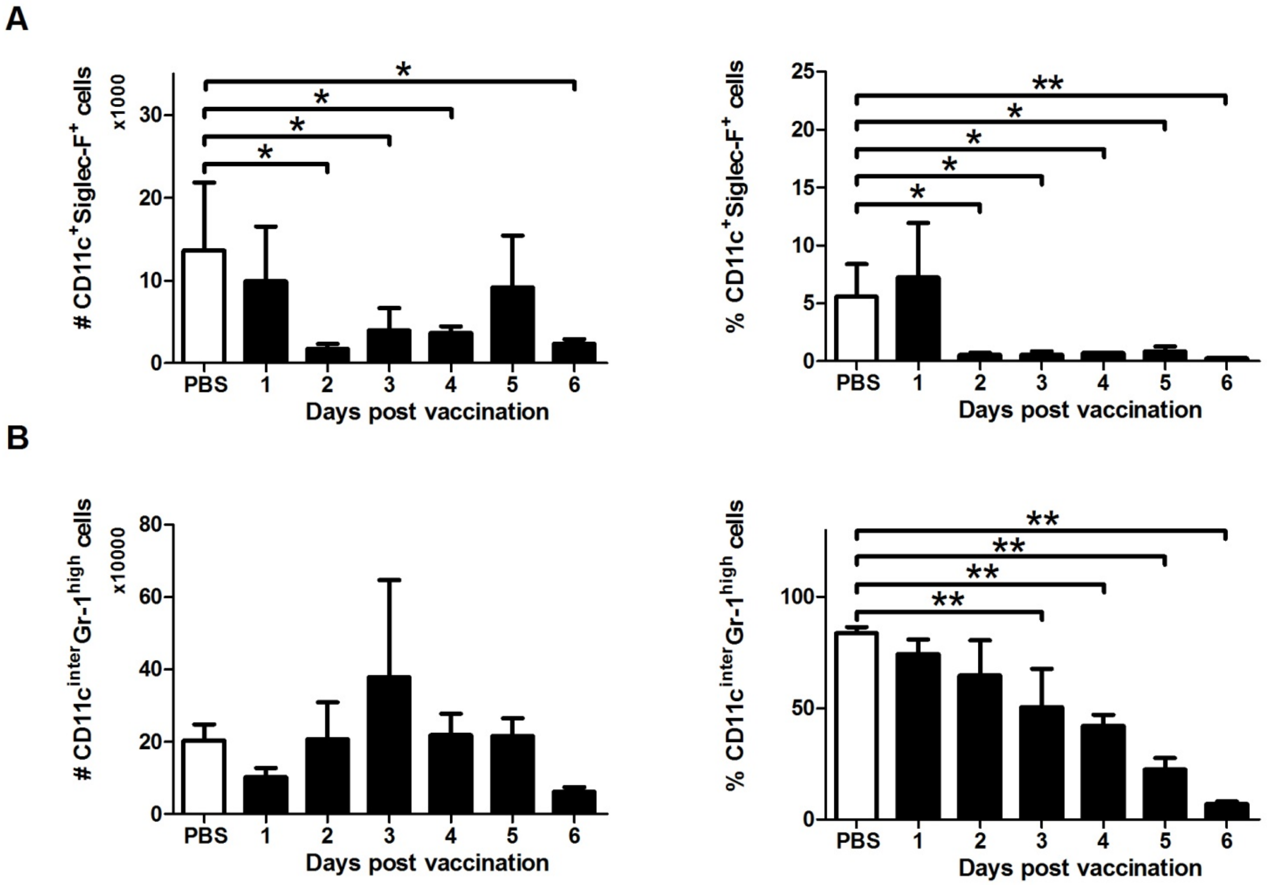

Supplementary Figure S4. Number and percentage of alveolar macrophages (AMs) and neutrophils after X-31ca vaccination. (**A and B**) Mice (n=4 per group) were vaccinated with 10^6 PFU of X-31ca, and BAL fluids were collected daily for 6 days. To investigate the number of and the percentage of AMs (**A**) and neutrophils (**B**), cells obtained from BAL were stained with fluorescence antibodies as presented in the Methods and analyzed by flow cytometry. ($^{*}P < 0.05$, $^{**}P < 0.01$ compared with the PBS control group)

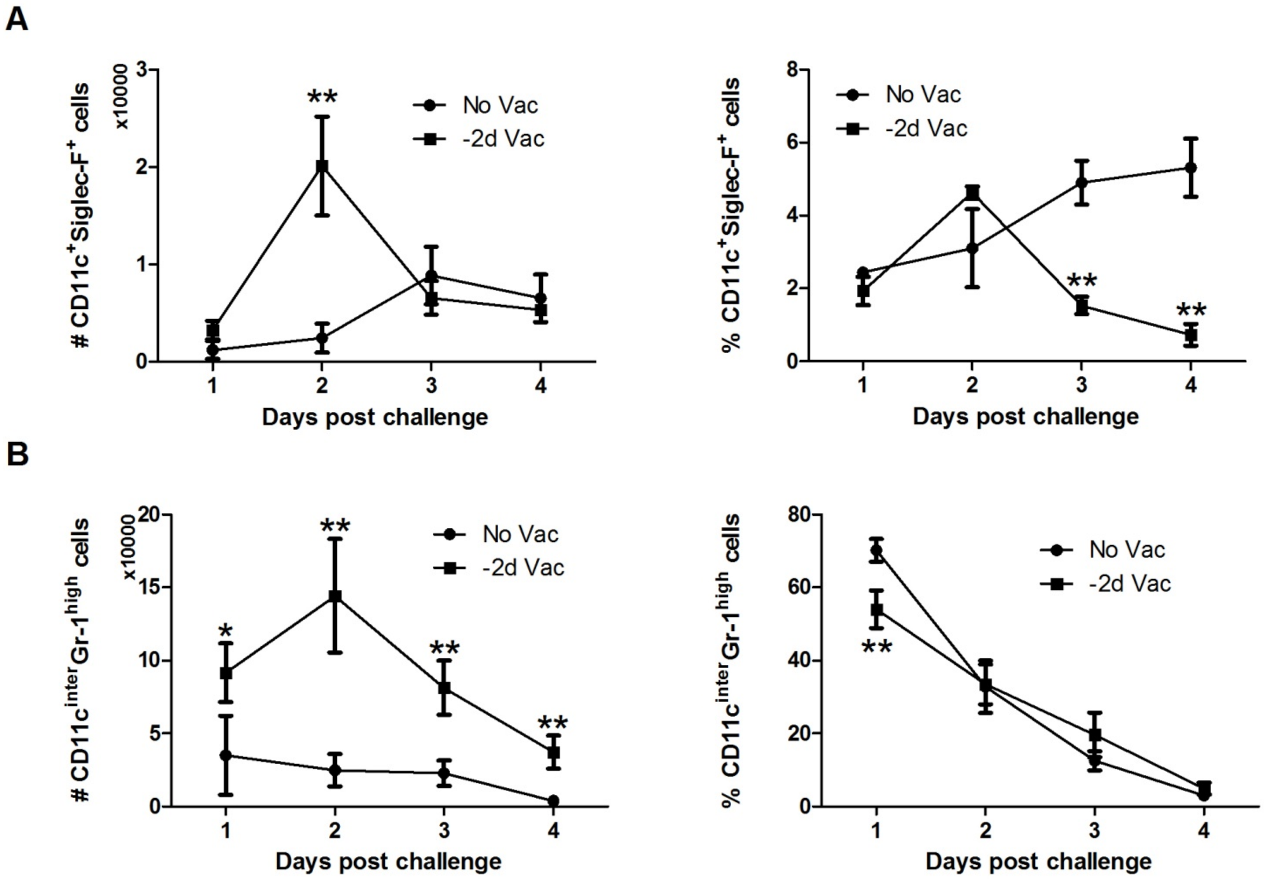

Supplementary Figure S5. Number and the percentage of alveolar macrophages (AMs) and neutrophils after X-31ca and RSV infection. (**A and B**) Mice (n=4 per group) were vaccinated with 10^6 PFU of X-31ca and challenged with 10^6 PFU of RSV A2 2 days later (-2d Vac). Non-vaccinated mice were infected with 10^6 PFU of RSV A2 only (No Vac). To investigate the number and the percentage of AMs (**A**) and neutrophils (**B**), BAL cells were obtained daily for 4 days after RSV challenge, and flow cytometry analysis was performed as in Supplementary Figure 4. ($^{*}P < 0.05$, $^{**}P < 0.01$ compared with the No Vac control group)

References

Schneider, C., Nobs, S.P., Heer, A.K., Kurrer, M., Klinke, G., van Rooijen, N., et al. (2014). Alveolar macrophages are essential for protection from respiratory failure and associated morbidity following influenza virus infection. *PLoS Pathog* 10(4)**,** e1004053. doi: 10.1371/journal.ppat.1004053.
